# Effect of processing on composition changes of selected spices

**DOI:** 10.1371/journal.pone.0176037

**Published:** 2017-05-01

**Authors:** Cai-Hua Jia, Jung-Ah Shin, Young-Min Kim, Ki-Teak Lee

**Affiliations:** 1 Key Laboratory of Environment Correlative Dietology (Ministry of Education), College of Food Science and Technology, Huazhong Agricultural University, Wuhan, Hubei, China; 2 Department of Food Science and Technology, Chungnam National University, Daejeon, Republic of Korea; Northwest Agriculture and Forestry University, CHINA

## Abstract

The present investigation was conducted to study the true retentions of α-tocopherol, tocotrienols and β-carotene in crown daisy, unripe hot pepper, onion, garlic, and red pepper as affected by various domestic cooking methods, those were, boiling, baking, stir-frying, deep-frying, steaming, roasting, and microwaving. Fatty acid compositions were determined by GC, and HPLC were used for quantification of α-tocopherol, tocotrienols, and β-carotene. True retentions of α-tocopherol in cooked foods were as follows: boiling (77.74–242.73%), baking (85.99–212.39%), stir-frying (83.12–957.08%), deep-frying (162.48–4214.53%), steaming (45.97–179.57%), roasting (49.65–253.69%), and microwaving (44.67–230.13%). Similarly for true retention of β-carotene were: boiling (65.69–313.75%), baking (71.46–330.16%), stir-frying (89.62–362.46%), deep-frying (178.22–529.16%), steaming (50.39–240.92%), roasting (73.54–361.47%), and microwaving (78.60–339.87%).

## Introduction

Spices, which are widely acceptable food adjuncts, affect flavor, pungency, antioxidant properties, and nutritional characteristics. The investigation of various nutritional components is important, especially the level change of nutrients caused by cooking treatments [1]. Cooking is necessary for most vegetables and causes positive or negative changes in the compositions or bioavailabilities of biochemicals, including those of different nutrients in vegetables [2, 3]. Information on the contents and true retentions of different nutrients in a series of spices is limited, although a number of studies have examined the effects of cooking treatments on fatty acid profiles and vitamin E or β-carotene levels in various foods [3–5]. Hence, determination of the nutrient contents of spices and their true retentions after cooking would contribute to salutary food preparation and possibly result in healthy cooking recommendation for the general public from the standpoint of food science.

Fatty acids, both straight-chain and branched-chain, have the ability to regulate lipid metabolism at different levels [6]. Dietary lipids are mainly hydrolyzed to free fatty acids and 2-monoglycerides in the stomach and small intestine, and afterwards these hydrolysates are absorbed and re-esterified to triglycerides or phospholipids in smooth endoplasmic reticulum [7]. Due to nutritional and health-related considerations, it was recommended that the consumption of fat, especially of saturated fatty acids (SFA) and trans-fatty acids (TFA), should be reduced [8, 9].

Vitamin E includes four tocopherols (α, β, γ, δ) and four tocotrienols (α, β, γ, δ), and is a well-known lipid-soluble antioxidant and free radical scavenger [10]. It is also believed to regulate enzymatic activity, to influence gene expression, and to inhibit platelet aggregation [11, 12]. Of these tocopherols and tocotrienols, α-tocopherol has the highest biological activity and is the most abundant form found in nature [13]. Furthermore, tocotrienols have greater antioxidant activities than tocopherols [14], and have been reported to inhibit smooth muscle cell proliferation to some extent [15].

The β-carotene is known to have provitamin A activity. The conjugated polyene of carotenoids allow it to absorb light or quench free radicals during photosynthesis [16]. It has been shown that β-carotene can prevent erythropoietic protoporphyria and protect from mitochondrial DNA mutation, a potential cause of carcinogenesis [17, 18].

In this study, the effects of different domestic cooking procedures, those are, boiling, baking, stir-frying, deep-frying, steaming, roasting and microwaving, on fatty acid profiles, vitamin E and β-carotene in five spices (i.e., crown daisy, unripe hot pepper, onion, garlic, and red pepper) are explored and discussed.

## Materials and methods

### Chemicals and reagents

Standard of fatty acid methyl esters (37 components FAME mix), BF_3_-methanol (10%, w/w), pyrogallol and α-tocopherol (≥96%, HPLC) were purchased from Sigma-Aldrich Co. (St. Louis, MO, USA). C11:0 triundecanoin as triglyceride internal standard was obtained from Nu-Chek Prep, Inc. (Elysian, MN, USA). β-carotene (≥95%, HPLC) was provided by Wako Pure Chemical Industries, Ltd. (Osaka, Japan). Anhydrous sodium sulfate and 2,6-Di-tert-butyl-4-methylphenol (BHT) were from Junsei Chemical Co., Ltd. (Tokyo, Japan). KOH, NaCl, diethyl ether, hexane, ethyl acetate, ethanol, chloroform, and petroleum ether were analytical grade and acquired from Daejung Chemicals & Metals Co., Ltd. (Shiheung, South Korea). HPLC grade of chloroform, hexane, methanol, and iso-propanol were obtained from Fisher scientific Korea Ltd. (Seoul, Republic of Korea).

### Collection and preparation of spices samples

Fresh and health (without damage) materials including crown daisy, unripe hot pepper, onion, garlic and red pepper were purchased from a local market (Jeonju, Jeollabuk-do, South Korea) and immediately washed using tap water as soon as they reached Korean international culinary school (Jeonju, Jeollabuk-do, South Korea). After removing the excessive surface water with paper towel and inedible parts, 5 spices samples were randomly selected by similar size, and then cut into small size or pieces (6 cm for crown daisy, 1 cm for unripe hot pepper and red pepper, 2 cm for onion, and 0.5 cm for garlic, respectively). After mixing well, all the 5 kinds of samples were cooked using 7 different treatments, including boiling, baking, stir-frying, deep-frying, steaming, roasting, and microwaving (as shown in Table 1). Preliminary experiments were implemented to simulate home-cooking and all cooking procedures were conducted at least three times.

**Table 1:** Conditions of different cooking procedures for 5 different spices.

**Cooking method**	**Spices**	**Weight of spices and** **other cooking mediums**	**Type of cooking equipment and cooking condition**
**Boiling**	**Crown daisy**	**2045 g, 3 L water**	**Induction heating, 1400 W (90 sec) after boiling. Drain water by colander**
	**Unripe hot pepper**	**2586 g, 3 L water**	**Induction heating, 1400 W (120 sec) after boiling. Drain water by colander**
	**Onion**	**2452 g, 3 L water**	**Induction heating, 1400 W (120 sec) after boiling. Drain water by colander**
	**Garlic**	**2264 g, 3 L water**	**Induction heating, 1400 W (240 sec) after boiling. Drain water by colander**
	**Red pepper**	**2020 g, 3 L water**	**Induction heating, 1400 W (300 sec) after boiling. Drain water by colander**
**Baking**	**Crown daisy**	**2070 g**	**Induction heating, 2500 W (180 sec) on pan**
	**Unripe hot pepper**	**2626 g**	**Induction heating, 1800 W (240 sec) on pan**
	**Onion**	**2506 g**	**Induction heating, 1800 W (120 sec) on pan**
	**Garlic**	**2318 g**	**Induction heating, 1200 W (120 sec) + 800 W (240 sec) on pan**
	**Red pepper**	**2294 g**	**Induction heating, 1800 W (240 sec) on pan**
**Stir-frying**	**Crown daisy**	**2360 g, 60 mL oil**	**Induction heating, 2500 W (150 sec) on pan, oil absorbed by paper towel**
	**Unripe hot pepper**	**2830 g, 60 mL oil**	**Induction heating, 1800 W (270 sec) on pan, oil absorbed by paper towel**
	**Onion**	**2740 g, 60 mL oil**	**Induction heating, 1800 W (120 sec) on pan, oil absorbed by paper towel**
	**Garlic**	**2438 g, 60 mL oil**	**Induction heating, 1400 W (180 sec) on pan, oil absorbed by paper towel**
	**Red pepper**	**2230 g, 60 mL oil**	**Induction heating, 1800 W (270 sec) on pan, oil absorbed by paper towel**
**Deep-frying**	**Crown daisy**	**2545 g, 3 L oil**	**Electric fryer, 170°C (60 sec), oil removed by colander and paper towel**
	**Unripe hot pepper**	**2896 g, 3 L oil**	**Electric fryer, 170°C (90 sec), oil removed by colander and paper towel**
	**Onion**	**2744 g, 3 L oil**	**Electric fryer, 170°C (180 sec), oil removed by colander and paper towel**
	**Garlic**	**2472 g, 3 L oil**	**Electric fryer, 170°C (210 sec), oil removed by colander and paper towel**
	**Red pepper**	**2362 g, 3 L oil**	**Electric fryer, 170°C (150 sec), oil removed by colander and paper towel**
**Steaming**	**Crown daisy**	**1820 g, 3 L water**	**Induction heating, 1400 W (120 sec) in steamer after water boiling**
	**Unripe hot pepper**	**1892 g, 3 L water**	**Induction heating, 1400 W (120 sec) in steamer after water boiling**
	**Onion**	**890 g, 3 L water**	**Induction heating, 1400 W (180 sec) in steamer after water boiling**
	**Garlic**	**1562 g, 3 L water**	**Induction heating, 1400 W (360 sec) in steamer after water boiling**
	**Red pepper**	**1266 g, 3 L water**	**Induction heating, 1400 W (360 sec) in steamer after water boiling**
**Roasting**	**Crown daisy**	**2373 g**	**Convection oven, 160°C (360 sec)**
	**Unripe hot pepper**	**2528 g**	**Convection oven, 160°C (420 sec)**
	**Onion**	**1912 g**	**Convection oven, 160°C (1200 sec)**
	**Garlic**	**2164 g**	**Convection oven, 160°C (420 sec)**
	**Red pepper**	**1994 g**	**Convection oven, 160°C (480 sec)**
**Microwaving**	**Crown daisy**	**2470 g**	**700 W microwave oven, (each 823 g crown daisy, 360 sec)**
	**Unripe hot pepper**	**2614 g**	**700 W microwave oven, (each 871 g unripe hot pepper, 270 sec)**
	**Onion**	**2848 g**	**700 W microwave oven, (each 950 g onion, 480 sec)**
	**Garlic**	**2222 g**	**700 W microwave oven, (each 741 g garlic, 420 sec)**
	**Red pepper**	**2202 g**	**700 W microwave oven, (each 734 g red pepper, 360 sec)**

The cooked products were cooled to room temperature and weighed. Afterwards, the samples were homogenized and stored at –20°C for further chemical analysis. Raw materials were treated similarly.

### Analytical procedure

#### Lipid extraction and fatty acids determination

The extraction of lipid was conformed to NLS procedure [19]. The homogenized samples were accurately weighed (1 g) into 50 mL vial with cap. One mL triglyceride internal standard solution (IS: C11:0 triundecanoin 5 mg/mL in iso-octane) and 2 mL pyrogallol solution (50 mg/mL in 95% ethanol) were added into the vial followed by 10.0 mL 8.3 M HCl. After mixing well, the vials were placed in water bath at 70–80°C for 1 h with moderate agitation speed (200 rpm). Vortex samples in 20 min interval and then 15 mL diethyl ether was added for extraction. After centrifugal treatment (2500 rpm, 3 min), the supernatant was dehydrated through anhydrous sodium sulfate column and transferred to new vial. Furthermore, 15 mL petroleum ether was used to extract lipid for another 2 times. All supernatant were collected and flushed with N_2_ and the residues were lipid extraction, which can be used to measure the fat content in samples.

In the meantime, the extracted lipid was subjected to total fatty acid composition analysis according to previous study [20].

#### Determination of moisture and fat contents

Moisture content was measured as weight variation according to previous study [2]. Triplicate of fresh or cooked samples (3–4 g) were placed in convection oven at 105°C for 16 h until constant weight. The fat content of samples was determined gravimetrically based on the lipid extraction as above.

#### Extraction procedure for vitamin E and β-carotene

The α-tocopherol, tocotrienols and β-carotene were extracted according to the methods of AOAC and NLS procedure [19, 21]. 5.0 g of samples was added to 10 mL 6% pyrogallol solution (in ethanol). After vortex 2 min, N_2_ flushing and sonication 10 min, 8 mL of 60% KOH solution (in distilled water) was added for saponification reaction. Then vortex 2 min and N_2_ flushing treatment were followed by the procedure of digestion at 75°C for 1 h in 100 rpm shaking water bath. Afterward, 20 mL of 2% NaCl solution (in distilled water) was added to extraction tube and 15 mL of the solution (hexane:ethyl acetate, 85:15, v/v, BHT 0.01%) were used to extract vitamin E and β-carotene for 3 times. The supernatant was collected, dehydrated with self-made anhydrous sodium sulfate column and transferred to 50 mL volumetric flask. Then additional extraction solution were put into volumetric flask to meet set point and final extraction solution was decanted into a 50 mL capped vial for –20°C storage. All samples were extracted in duplicate and precautions (i.e., samples in ice, darkened room, little exposure, and N_2_ flushing, etc.) were taken to protect the nutrients.

#### Determinations of α-tocopherol and tocotrienols

The quantification of α-tocopherol and tocotrienols followed the previous study [22]. 5 mL of extraction solution was taken out and flushed with N_2_. Afterward, 2.5 mL hexane (HPLC grade) was used to dissolve the prepared samples. Then the analytical solution was filtered with 0.5 μm hydrophobic membrane and injected immediately into the high-performance liquid chromatography.

The HPLC instrument included LiChrosorb Diol Column (5 μm, LC 100 × 3 mm i.d., Chromapack, Raritan, N.J., USA) with FLD detector (Exλ = 290 nm, Emλ = 330 nm). A mixture of hexane and iso-propanol in the ratio of 99.4:0.6 (v/v) was served as the mobile phase solvent at a flow rate of 1.0 mL/min and the analysis time was 60 min. In addition, stock solutions of α-, β-, γ-, and δ-tocopherol standard with 0.1, 0.5, 1, 5, 10, 20, 40 μg/mL were used for quantification. Results were presented as mg/100 g and μg/100 g wet weight of raw and cooked samples for α-tocopherol and tocotrienols.

#### Determination of β-carotene

Quantification of β-carotene was conducted with HPLC according to NLS procedure [19]. Ten mL of extraction solution were prepared and flushed with N_2_. Afterwards, 1 mL CHCl_3_ (HPLC grade) was added to dissolve the residues and 20 μL aliquot of analytical solution was injected into HPLC immediately after filtering the solution with 0.5 μm hydrophobic membrane.

**Fig 1:**
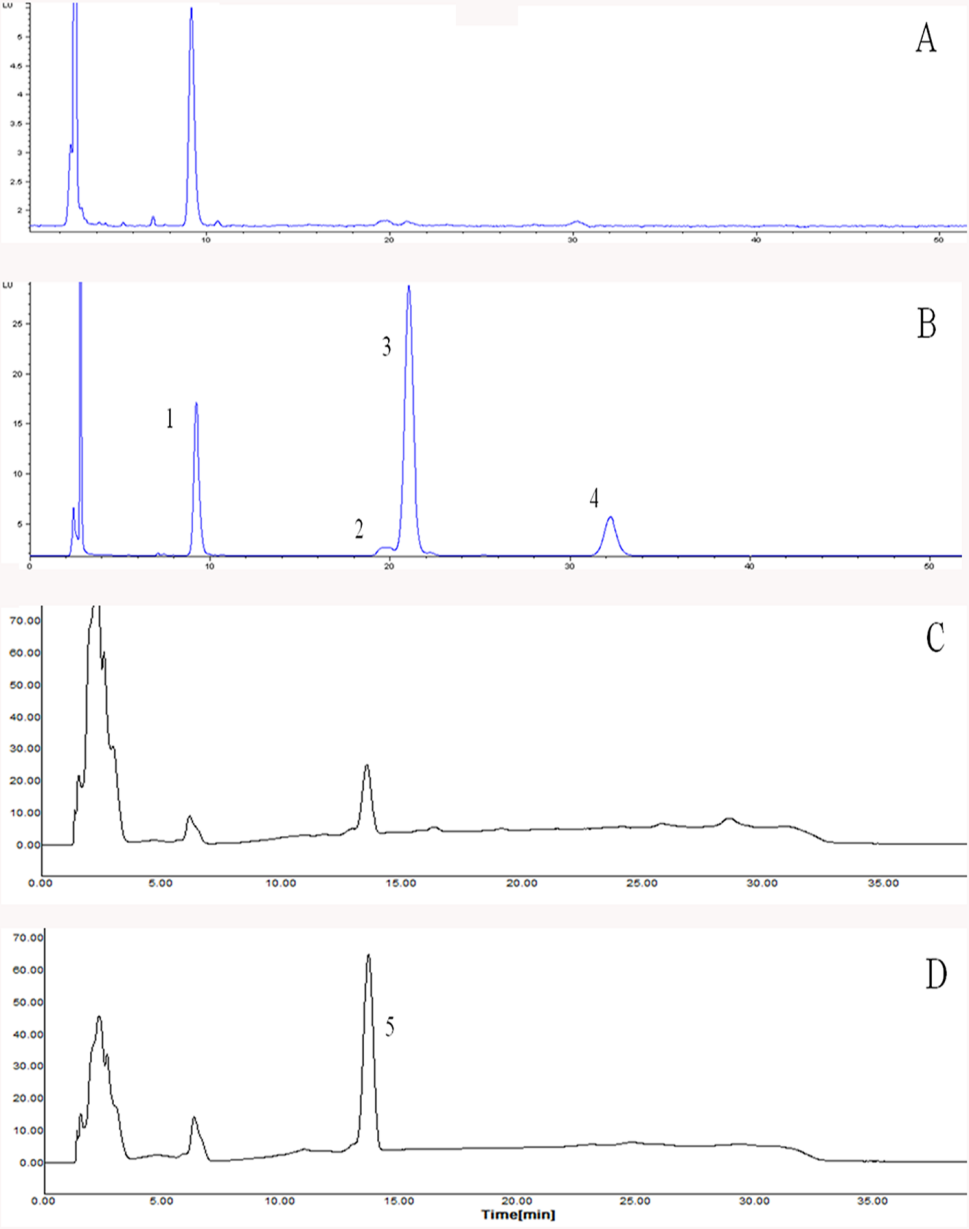
Chromatogram of tocopherols (A: raw red pepper, B: deep-fried red pepper) and β-carotene (C: raw red pepper, D: deep-fried red pepper). Peak identification: (1) α-tocopherol, (2) β-tocopherol, (3) γ-tocopherol, (4) δ-tocopherol, (5) β-carotene.

The high-performance liquid chromatography was equipped with Yonglin SP930D dual pump (Yonglin, Anayang, Korea) and Nova-Pak C18 column (4 μm, 150 × 3.9 mm i.d., Waters, Milford, M.A., USA) followed by UV detector (set at 450 nm). The mobile phase contained solvent A (ACN:MeOH:DCM, 70:10:30, v/v/v) and solvent B (ACN:MeOH:DCM, 75:20:5, v/v/v) at a flow rate of 1.0 mL/min and the run time was 40 min. The solvent gradient was as followed: 100% B for 3.5 min, change to 100% A during 18.5 min, hold for 6.5 min, change to 100% B with 1.5 min, hold for 10 min. The determination was carried out using external calibration curves, which were made by 0.1, 0.5, 1, 5, 10, 20, 40 μg/mL of β-carotene standard. Results were showed as μg/100 g wet weight of raw and cooked vegetables. The chromatogram of 4 tocopherols and β-carotene in red pepper were characterized in Fig 1.

### True retentions of nutrients

The true retention was calculated as the following formula, according to previous study [23, 24].

$$True\;retention\;(\%)=\frac{nutrient\;content\;(\frac{g}{100g})\;of\;cooked\;vegetable\;\times \;g\;of\;cooked\;vegetable}{nutrient\;content\;(\frac{g}{100g})\;of\;raw\;vegetable\;\times \;g\;of\;raw\;vegetable}\times 100$$

The percent variation due to cooking treatment is given as below:

$$Percent\;variation\;(\%)=\frac{nutrient\;content\;of\;cooked\;vegetatble-nutrient\;content\;of\;raw\;vegetable}{nutrient\;content\;of\;raw\;vegetable}\times 100$$

### Statistical analysis

The results were presented as the means ± SD of three measurements. Statistical analysis was performed using SAS (SAS Institute Cary, NC, USA) software. Analysis of variance (ANOVA) was carried out and means comparisons was performed with Duncan’s multiple-range test. The statistical significance for all tests was set at a level of *p* < 0.05.

## Results and discussion

### Moisture and fat contents and true retention of fat

**Fig 2:**
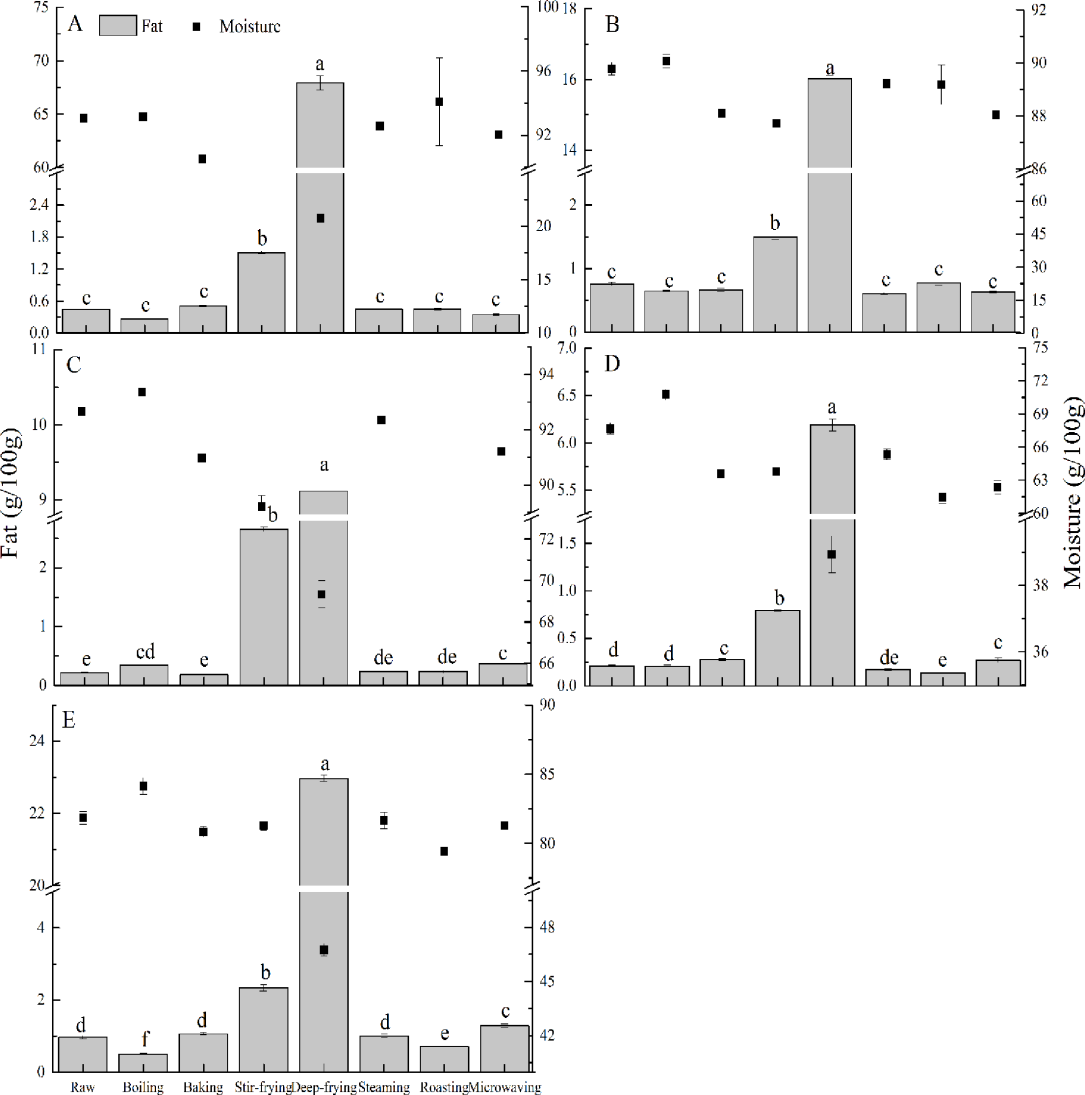
Fat (histogram) and moisture content (■) of crown daisy (A), unripe hot pepper (B), onion (C), garlic (D) and red pepper (E). Values are mean ± SD (n ≥ 3). Bars of histograms (fat content) with same letters are not significantly different (p > 0.05).

Moisture and fat contents are shown in Fig 2. Deep-frying was found to result in lower moisture contents and higher fat levels than other treatments. Of the 5 spices investigated, moisture content ranges except deep-frying were: crown daisy (89.93–94.07 g/100 g), unripe hot pepper (87.71–90.06 g/100 g), onion (88.45–93.35 g/100 g), garlic (61.44–70.75 g/100g) and red pepper (79.44–84.16 g/100 g), whereas that of deep-frying were: 20.71, 69.37, 69.34, 38.94 and 46.76 g/100 g, respectively. These results are in accord with those of previous studies [25, 26]. With respect to fat content, levels in most cooked spices were similar to those of raw ones, excepting some significant increases for deep-frying and stir-frying (*p* < 0.05). Fat contents of fresh spices ranged from 0.21 to 0.97 g/100 g while those of non-fried products (boiling, baking, steaming, roasting and microwaving) was shown as 0.14–1.29 g/100 g. However, fat contents of deep-fried and stir-fried samples were up to 6.19–67.92 and 0.79–2.65 g/100 g, respectively.

**Fig 3:**
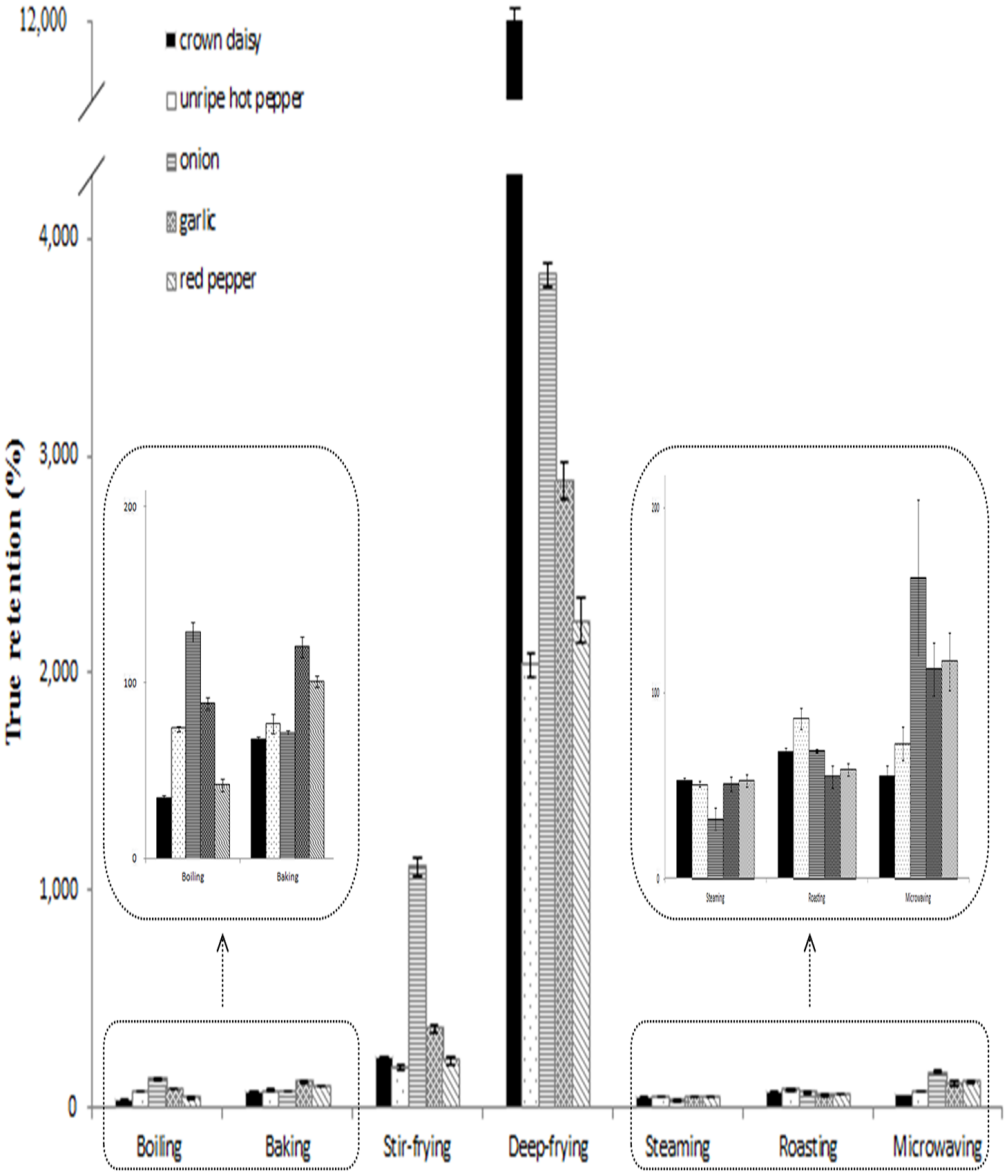
True retention of fat for 5 spices cooked in different procedures.

Fig 3 shows true retentions of fat for five spices prepared using different cooking methods. Less than 162% of true retention of fat was found in non-frying cooking methods except for deep-frying and stir-frying, which gave values of 186.50–11168.66%. The reason why deep-frying produced high fat content and true retention is probably due to not only dehydration but also oil absorption from frying medium [26]. Furthermore, the remarkable results for deep-fried crown daisy can be explained by its leafy morphological structure in comparison to other spices.

### Main fatty acids distribution

The fatty acid profiles of raw and cooked spices were listed in Table 2. Quantitatively, amounts of fatty acids in raw spices were low, and only marginal changes in fatty acid composition were observed for five non-frying cuisines. However, apparent increases in Σ SFA (total saturated fatty acids), Σ UFA (total unsaturated fatty acids), Σ TFA (total trans-fatty acids) and Σ FA (total fatty acids) were determined for the 5 spices when deep-fried, followed by moderate increases when stir-fried. For example, the content of Σ FA from deep-frying were 57.69 g/100 g having 83.78% of unsaturated fatty acids in crown daisy, whereas that of raw and stir-fried crown daisy were 0.21 g/100 g and 0.89 g/100 g, respectively. Furthermore, trans-fatty acids were only found in two fried crown daisy. Even though trans-fatty acids were not detected in raw crown daisy, Σ TFA were 0.01 and 0.44 g/100 g for stir-fried and deep-fried products. Similar patterns were also observed in other spices. Differences between fatty acid distributions after deep-frying and stir-frying can be attributed to their cooking temperature and fat absorption from cooking medium. The pervious study mentioned that increases in oleic acid and linoleic acid after deep-frying potatoes, hake, and sardines were mainly attributable to the absorption of olive oil, which was used as frying medium [26].

**Table 2:** Total fatty acid profiles of 5 spices and their cooked products^a^.

	**Σ SFA**^b^ **(g/100 g)**	**Σ UFA**^c^ **(g/100 g)**	**Σ TFA**^d^ **(g/100 g)**	**Σ FA**^e^ **(g/100 g)**
	**Crown daisy**			
**Raw**	**0.03 ± 0.002**	**0.17 ± 0.03**	**0.00 ± 0.00**	**0.21 ± 0.03**
**Boiling (1.5 min)**	**0.04 ± 0.01**	**0.23 ± 0.04**	**0.00 ± 0.00**	**0.28 ± 0.04**
**Baking (3)**	**0.04 ± 0.003**	**0.26 ± 0.05**	**0.00 ± 0.00**	**0.30 ± 0.05**
**Stir-frying (2.5)**	**0.14 ± 0.03**	**0.74 ± 0.17**	**0.01 ± 0.001**	**0.89 ± 0.21**
**Deep-frying (1)**	**8.92 ± 0.17**	**48.33 ± 0.21**	**0.44 ± 0.08**	**57.69 ± 0.22**
**Steaming (2)**	**0.09 ± 0.11**	**0.09 ± 0.03**	**0.00 ± 0.00**	**0.18 ± 0.08**
**Roasting (6)**	**0.03 ± 0.01**	**0.18 ± 0.05**	**0.00 ± 0.00**	**0.21 ± 0.05**
**Microwaving (6)**	**0.03 ± 0.01**	**0.20 ± 0.06**	**0.00 ± 0.00**	**0.24 ± 0.07**
	**Unripe hot pepper**			
**Raw**	**0.08 ± 0.02**	**0.36 ± 0.10**	**0.00 ± 0.001**	**0.43 ± 0.12**
**Boiling**	**0.08 ± 0.02**	**0.37 ± 0.09**	**0.00 ± 0.00**	**0.44 ± 0.11**
**Baking**	**0.08 ± 0.02**	**0.42 ± 0.08**	**0.00 ± 0.00**	**0.50 ± 0.10**
**Stir-frying**	**0.18 ± 0.07**	**0.96 ± 0.35**	**0.01 ± 0.003**	**1.14 ± 0.42**
**Deep-frying**	**2.21 ± 0.21**	**11.89 ± 1.04**	**0.12 ± 0.01**	**14.22 ± 1.22**
**Steaming**	**0.04 ± 0.01**	**0.26 ± 0.13**	**0.00 ± 0.00**	**0.31 ± 0.13**
**Roasting**	**0.05 ± 0.004**	**0.25 ± 0.01**	**0.00 ± 0.00**	**0.30 ± 0.01**
**Microwaving**	**0.09 ± 0.02**	**0.46 ± 0.10**	**0.00 ± 0.00**	**0.56 ± 0.12**
	**Onion**			
**Raw**	**0.02 ± 0.002**	**0.01 ± 0.003**	**0.00 ± 0.00**	**0.03 ± 0.004**
**Boiling**	**0.02 ± 0.01**	**0.06 ± 0.04**	**0.01 ± 0.02**	**0.09 ± 0.05**
**Baking**	**0.11 ± 0.17**	**0.03 ± 0.01**	**0.01 ± 0.01**	**0.15 ± 0.18**
**Stir-frying**	**0.40 ± 0.06**	**2.14 ± 0.26**	**0.02 ± 0.01**	**2.56 ± 0.32**
**Deep-frying**	**1.22 ± 0.25**	**6.53 ± 1.14**	**0.09 ± 0.02**	**7.84 ± 1.38**
**Steaming**	**0.02 ± 0.01**	**0.05 ± 0.02**	**0.00 ± 0.001**	**0.07 ± 0.02**
**Roasting**	**0.02 ± 0.01**	**0.03 ± 0.01**	**0.00 ± 0.00**	**0.05 ± 0.01**
**Microwaving**	**0.01 ± 0.002**	**0.03 ± 0.004**	**0.00 ± 0.00**	**0.04 ± 0.01**
	**Garlic**			
**Raw**	**0.03 ± 0.01**	**0.07 ± 0.02**	**0.00 ± 0.001**	**0.10 ± 0.02**
**Boiling**	**0.02 ± 0.001**	**0.07 ± 0.02**	**0.00 ± 0.001**	**0.10 ± 0.02**
**Baking**	**0.03 ± 0.01**	**0.08 ± 0.03**	**0.00 ± 0.00**	**0.11 ± 0.03**
**Stir-frying**	**0.12 ± 0.004**	**0.63 ± 0.10**	**0.01 ± 0.001**	**0.76 ± 0.11**
**Deep-frying**	**1.04 ± 0.22**	**5.43 ± 0.94**	**0.06 ± 0.002**	**6.53 ± 1.15**
**Steaming**	**0.02 ± 0.01**	**0.07 ± 0.03**	**0.00 ± 0.00**	**0.09 ± 0.04**
**Roasting**	**0.03 ± 0.01**	**0.08 ± 0.03**	**0.00 ± 0.001**	**0.11 ± 0.04**
**Microwaving**	**0.03 ± 0.01**	**0.08 ± 0.04**	**0.00 ± 0.00**	**0.11 ± 0.04**
	**Red pepper**			
**Raw**	**0.05 ± 0.01**	**0.12 ± 0.01**	**0.01 ± 0.02**	**0.18 ± 0.05**
**Boiling**	**0.05 ± 0.002**	**0.18 ± 0.05**	**0.00 ± 0.00**	**0.23 ± 0.05**
**Baking**	**0.08 ± 0.02**	**0.22 ± 0.06**	**0.00 ± 0.00**	**0.30 ± 0.08**
**Stir-frying**	**0.31 ± 0.09**	**1.43 ± 0.41**	**0.01 ± 0.001**	**1.75 ± 0.50**
**Deep-frying**	**3.23 ± 0.62**	**16.56 ± 2.36**	**0.16 ± 0.02**	**19.95 ± 2.96**
**Steaming**	**0.10 ± 0.03**	**0.33 ± 0.12**	**0.00 ± 0.00**	**0.44 ± 0.15**
**Roasting**	**0.08 ± 0.02**	**0.21 ± 0.07**	**0.00 ± 0.00**	**0.29 ± 0.09**
**Microwaving**	**0.09 ± 0.03**	**0.29 ± 0.11**	**0.00 ± 0.00**	**0.39 ± 0.14**

^a^ Values are mean ± SD of three measurements.

^b^ Σ SFA (total saturated fatty acids) includes C 4:0, C6:0, C8:0, C10:0, C12:0, C14:0, C16:0, C18:0 and C20:0.

^c^ Σ UFA (total unsaturated fatty acids) meant the sum of C14:1, C16:1, C18:1(n-9), C18:1(n-7), C18:2(n-6), C18:3(n-6), C20:1, C18:3(n-3), C20:2, C20:3, C20:4, C20:5, C22:5, C22:6.

^d^ Σ TFA (total trans-fatty acids) contains C18:1t, C18:2t and C18:3t.

^e^ Σ FA (total fatty acids) are the sum of SFA, UFA and TFA.

### Contents and true retentions of α-tocopherol and total tocotrienols

**Fig 4:**
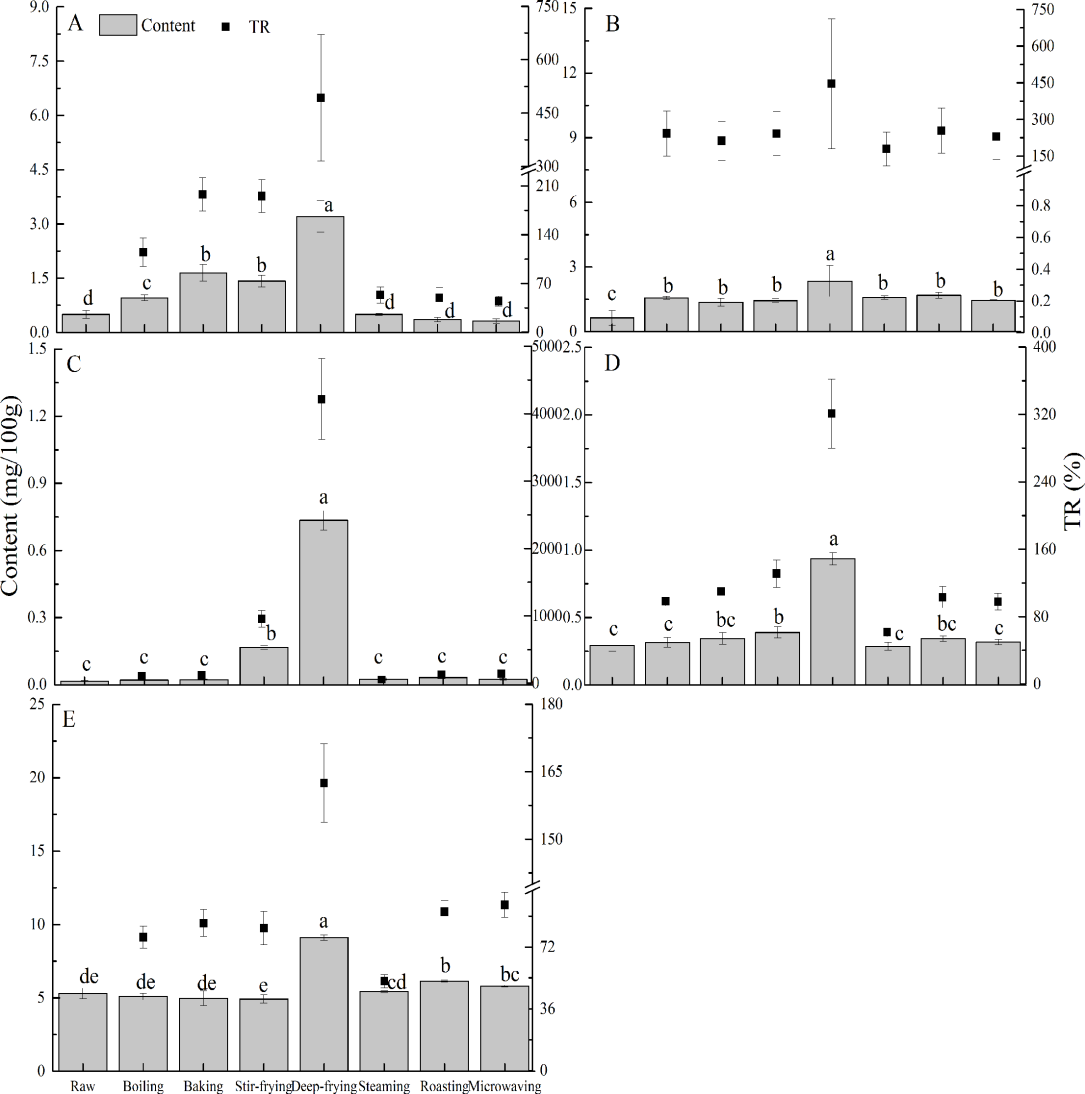
The content of α-tocopherol (histogram) and related true retention (TR) (■) for crown daisy (A), unripe hot pepper (B), onion (C), garlic (D) and red pepper (E). Values are mean ± SD of three measurements. Bars of histograms (α-tocopherol content) with same letters are not significantly different (p > 0.05).

The contents and true retentions of α-tocopherol in the 5 spices cooked in different methods are shown in Fig 4. Interestingly, α-tocopherol showed somewhat different patterns in the five spices. For crown daisy, boiling, baking, stir-frying, and deep-frying led to significant increase in α-tocopherol content (*p* < 0.05), although steaming, roasting, and microwaving did not distinctly change α-tocopherol levels, providing low true retention value. True retentions of α-tocopherol after 7 cooking methods in crown daisy ranged 44.67–493.01%, and deep-frying showed the maximum among them. In case of unripe hot pepper, all cooking methods induced more than 100% true retention of α-tocopherol in which deep-frying was the highest value (446.24%).

Although different true retentions of α-tocopherol for prepared garlic were found, deep-frying also gave the highest value of 320.98% compared to other cooking methods. In addition, it was found that the true retention of α-tocopherol for red pepper were less than 100% for most preparation except for deep-frying, showing 162.48%. Previous studies on the effects of cooking on α-tocopherol content in fresh red pepper reported that boiling, stewing, steaming and pressure steaming did not induce significant changes [3]. In the present study, analogous cooking methods produced similar results, whereas deep-frying, roasting and microwaving significantly improved the α-tocopherol content in red pepper (*p* < 0.05). The α-tocopherol contents for fresh, boiled, baked, steamed, roasted and microwaved onion were 0.02, 0.02, 0.02, 0.03, 0.03 and 0.02 mg/100 g, respectively, comparing with those of stir-frying (0.17 mg/100 g) and deep-frying (0.73 mg/100 g). α-Tocopherol is primarily found in chloroplasts and its levels gradually increase as fruits mature [27], and thus, the α-tocopherol level in red pepper is greater than that in unripe hot pepper. Besides, the main reason for more than 100% true retention of α-tocopherol in the 5 spices after deep-frying could have been due to the absorption of deep-frying oil by spices, since normal commercial frying oil contains additional vitamin E. Absorption could be proven by examining the fatty acid profiles of cooked and raw samples. Also, further analyses found the most significant interaction between treatment and vegetable for the determination of α-tocopherol (*p* < 0.01). It indicated that cooking processing and spices work together, making more significant impact on the true retention of α-tocopherol.

In addition, the measurement of total tocotrienols was conducted. Tocotrienols were not detected in any treated crown daisy, onion, or garlic, except for α-tocotrienol in unripe hot pepper and red pepper (Table 3). Until now, few studies have examined changes in the **α-**tocotrienol contents of vegetables caused by different cooking methods. It was found that deep-frying resulted in more than 100% true retention of **α-**tocotrienol in unripe hot pepper and red pepper, which is similar to that observed for α-tocopherol. As stated above, percent variation is the relative change of nutrients level in cooked products as compared with raw materials. For unripe hot pepper, percentage changes of **α-**tocotrienol caused by boiling, baking, stir-frying, deep-frying, steaming, roasting and microwaving were 13.71%, 21.63%, –1.13%, 50.33%, –3.37%, 10.84% and –10.79%, respectively, whereas corresponding values for red pepper were –5.49%, –25.08%, 9.06%, 44.11%, –11.20%, 4.34% and –2.64%. Such differences in percent variation were attributed to variety of vegetable and cooking methods. Furthermore, no significant interaction between treatment and vegetable was found (*p* > 0.05).

**Table 3:** Effect of different cooking methods on the content, true retention and percent variation of α-tocotrienol in unripe hot pepper and red pepper.

**Spices**	**Method**	**α-tocotrienol (μg/100 g)**^a^	**True retention (%)**^b^	**Percent variation (%)**^b^
**Unripe hot pepper**	**Raw**	**12.02 ± 5.24**^**A**^	–	–
	**Boiling**	**11.03 ± 1.76**^**A**^	**98.02**	**13.71**
	**Baking**	**13.73 ± 4.24**^**A**^	**106.46**	**21.63**
	**Stir-frying**	**10.40 ± 2.91**^**A**^	**93.27**	**–1.13**
	**Deep-frying**	**13.06 ± 5.15**^**A**^	**145.12**	**50.33**
	**Steaming**	**10.04 ± 1.86**^**A**^	**60.95**	**–3.37**
	**Roasting**	**10.64 ± 1.24**^**A**^	**93.40**	**10.84**
	**Microwaving**	**8.94 ± 2.42**^**A**^	**77.73**	**–10.79**
**Red pepper**	**Raw**	**22.71 ± 3.25**^**A**^	–	–
	**Boiling**	**20.90 ± 5.14**^**A**^	**76.37**	**–5.49**
	**Baking**	**16.75 ± 5.93**^**A**^	**68.75**	**–25.08**
	**Stir-frying**	**24.15 ± 2.50**^**A**^	**97.28**	**9.06**
	**Deep-frying**	**31.34 ± 18.80**^**A**^	**136.16**	**44.11**
	**Steaming**	**19.65 ± 7.69**^**A**^	**44.97**	**–11.20**
	**Roasting**	**22.99 ± 7.36**^**A**^	**83.22**	**4.34**
	**Microwaving**	**21.37 ± 7.79**^**A**^	**85.75**	**–2.64**

^a^ Values are mean ± SD of three measurements.

^b^ True retention and percent variation were calculated using mean values of nutrient content.

^A^ The content of α-tocotrienol with same letters are not significantly different (*p* > 0.05).

### Content and true retention of β-carotene

**Fig 5:**
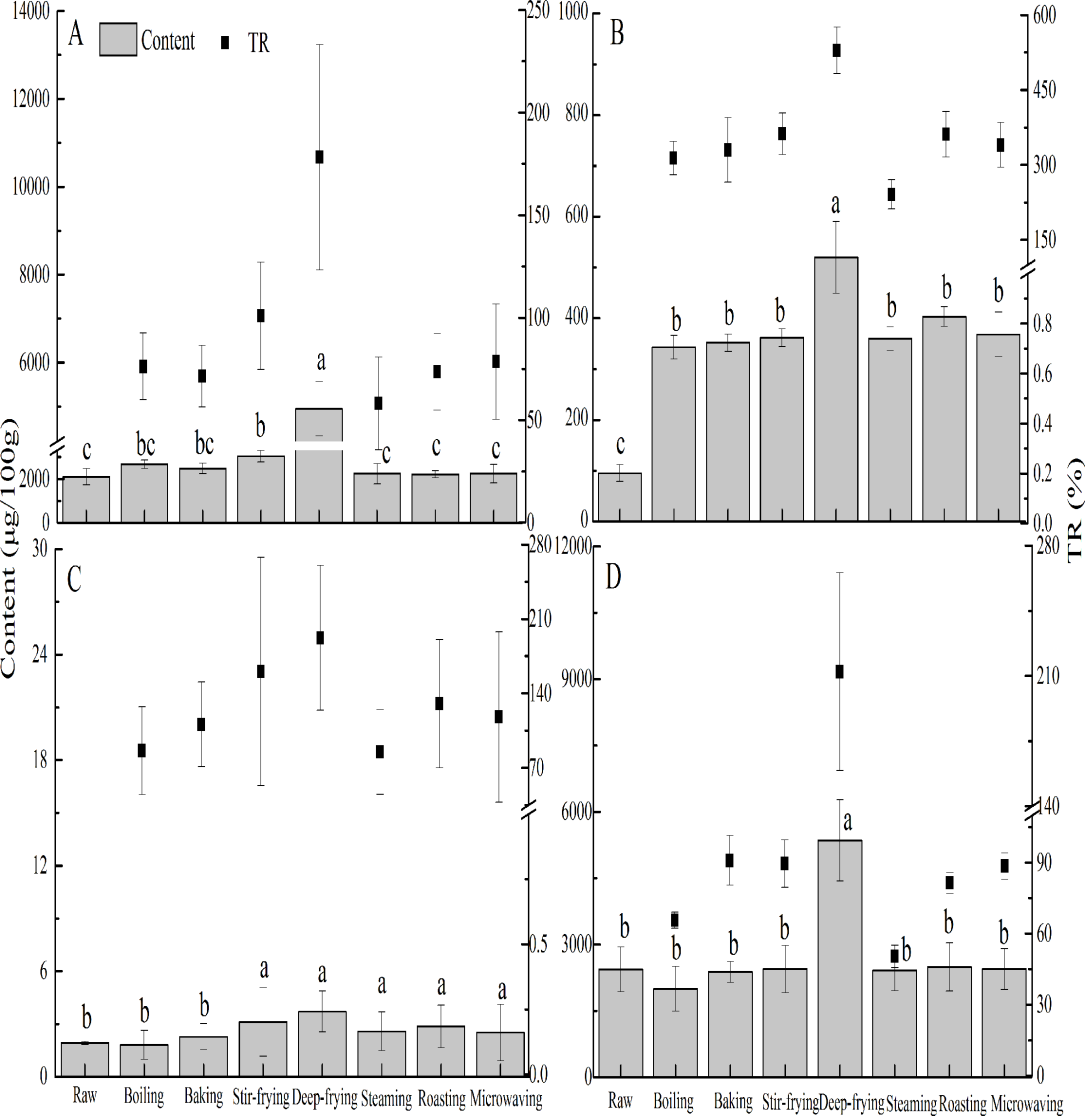
The content of β-carotene (histogram) and related true retention (TR) (■) of crown daisy (A), unripe hot pepper (B), garlic (C) and red pepper (D). Values are presented as mean ± SD of three measurements. Bars of histograms (β-carotene content) with same letters are not significantly different (p > 0.05).

Generally β-carotene exists in the oil droplets of orange or red fruit chromoplasts [28, 29]. The contents and true retentions of β-carotene for the 5 spices differed for the seven cooking methods (Fig 5). In crown daisy and red pepper, β-carotene contents ranged from 1998.34 to 5353.32 μg/100 g with less than 100% true retention by boiling, baking, stir-frying, steaming, roasting and microwaving. However, true retentions were greater than 100% after deep-frying (178.22% for crown daisy and 212.37% for red pepper). Moreover, in red pepper, cooking methods did not much change β-carotene content, except for deep-frying with significant increase (*p* < 0.05). The results of the present study were somewhat different from previous study [3], in which cooking (i.e., boiling, stewing, steaming, and pressure steaming) had negative effects on β-carotene content in fresh red pepper. The inconsistency could be due to different cooking parameters (i.e., time, temperature, material to water ratio, cultivation location, etc.) even in similar cooking methods. Among the cooking methods, deep-frying led to higher true retention of β-carotene in red pepper, which might be due to cell wall destruction and greater β-carotene extractability in cooked samples [2]. In the present study, the amounts of β-carotene observed in raw and cooked unripe hot pepper was ranged from 95.36 to 519.96 μg/100 g. Meanwhile, more than 100% true retention of β-carotene was found in all cooking methods with a maximum of 529.16% in deep-frying. No β-carotene was detected in onion. Finally, β-carotene contents were below 3.71 μg/100 g for raw and prepared garlic, and corresponding true retentions was less than 192.12% (deep-fried garlic). According to previous study [29], β-carotene was linked to chlorophyll-binding proteins, which inhibits its accessibility in the vegetable matrix. Cooking softens vegetable tissues and increases the extractability of β-carotene, and thus, probably increases its bioavailability. The variable true retentions of β-carotene by unripe hot pepper and garlic during cooking could be related to vegetables varieties, methods of treatment, and interactions between the two [30]. It could be proven by the most significant interaction between treatment and spices for the determination of β-carotene (*p* < 0.01). In a previous study, it was pointed out that more than 100% true retention of carotenoid in cooked products did not indicate a true increment in level, but rather reflected the difficulties associated with determining nutrient retention accurately [31].

### Percent variation in fat, moisture, α-tocopherol, and β-carotene contents

The effects of seven cooking methods on the percent variation of fat, moisture, α-tocopherol, and β-carotene contents are summarized in Table 4. Regarding the rate of changes in fat content, deep-frying and stir-frying showed increases of 97.70–15259.66%. There were not distinct changes in moisture contents depending on six cooking methods, except that apparent decreases were observed from deep-frying (–77.73% for crown daisy, –22.73% for unripe hot pepper, –25.17% for onion, –42.58% for garlic and –42.87% for red pepper). With respect to percent variation of α-tocopherol contents in the 5 spices, amount of α-tocopherol increased in unripe hot peppers when cooked using seven methods while levels in other spices are variable in this study.

**Table 4:** Effect of different cooking methods on the percent variation of fat, moisture, α-tocopherol and β-carotene contents in 5 spices^a^.

	**Boiling**	**Baking**	**Stir-frying**	**Deep-frying**	**Steaming**	**Roasting**	**Microwaving**
	**Rate of fat content change (%)**						
**Crown daisy**	**–40.98**^**b**^	**14.31**^**c**^	**241.08**	**15259.66**	**1.51**	**0.75**	**–21.63**
**Unripe hot pepper**	**–14.30**	**–12.57**	**97.70**	**2009.24**	**–19.85**	**2.32**	**–16.61**
**Onion**	**57.74**	**–14.28**	**1118.19**	**4087.90**	**8.24**	**8.06**	**70.51**
**Garlic**	**–2.67**	**29.67**	**273.73**	**2818.35**	**–18.69**	**–36.48**	**27.02**
**Red pepper**	**–48.41**	**9.77**	**141.23**	**2269.16**	**3.78**	**–26.48**	**32.86**
	**Rate of moisture content change (%)**						
**Crown daisy**	**0.09**	**–2.73**	**–3.34**	**–77.73**	**–0.52**	**–1.57**	**–1.11**
**Unripe hot pepper**	**0.32**	**–1.87**	**–2.30**	**–22.73**	**–0.63**	**–0.57**	**–1.94**
**Onion**	**0.75**	**–1.82**	**–3.71**	**–25.17**	**–0.33**	**–4.54**	**–1.57**
**Garlic**	**4.52**	**–6.02**	**–5.95**	**–42.58**	**–3.48**	**–9.24**	**–7.87**
**Red pepper**	**2.83**	**–1.23**	**–0.69**	**–42.87**	**–0.21**	**–2.94**	**–0.59**
	**Rate of α-tocopherol content change (%)**						
**Crown daisy**	**96.62**	**234.88**	**189.90**	**578.01**	**2.99**	**–26.76**	**–36.71**
**Unripe hot pepper**	**181.59**	**142.64**	**157.02**	**362.26**	**184.73**	**201.05**	**164.11**
**Onion**	**28.26**	**43.71**	**947.90**	**4507.72**	**54.95**	**98.59**	**47.13**
**Garlic**	**8.28**	**18.49**	**34.43**	**224.62**	**–1.37**	**19.04**	**10.09**
**Red pepper**	**–3.79**	**–6.29**	**–6.82**	**71.98**	**2.71**	**16.15**	**9.64**
	**Rate of β-carotene content change (%)**						
**Crown daisy**	**30.43**	**20.82**	**49.56**	**145.09**	**11.74**	**8.47**	**11.37**
**Unripe hot pepper**	**263.98**	**277.19**	**284.24**	**448.17**	**282.02**	**328.95**	**290.05**
**Garlic**	**–5.10**	**19.58**	**64.67**	**94.30**	**35.91**	**50.41**	**32.52**
**Red pepper**	**–18.70**	**–0.92**	**0.47**	**124.78**	**–0.49**	**2.08**	**0.54**

^a^ Percent variation of different nutrients were calculated using mean values of corresponding nutrients content.

^b^(–): decrease of nutrients contents in cooked products.

^c^ increase of nutrients contents in cooked products.

The percent variations of α-tocopherol contents in cooked unripe hot pepper was more than 142.64%, but those of other spices was between –36.71 to 4507.72 percent. Finally, levels of β-carotene in unripe hot pepper were increased by all cooking methods, whereas levels in other cooked samples varied widely. The percent variation for cooked unripe hot pepper was ranged from 263.98 to 448.17% while those of other cooked spices were between –18.70 and 145.09%. Both of percent variations of α-tocopherol and β-carotene contents were more than 100% only in cooked unripe hot pepper, and the percent variation of four nutrients (fat, moisture, α-tocopherol, and β-carotene) in spices hereby concurred with corresponding nutrients determinations (Figs 2, 4 and 5). As has been previously reported, such variability was probably related to dehydration, softening or destruction of plant cell matrix, and leaching of nutrients to cooking medium [32]. It would appear that the positive value of percent variation suggests the net increase of nutrients, and vice versa.

As percent variation only explain the concentration changes owing to leaching nutrients to the cooking medium or to absorbing water or fat into food products, percent variation can be corrected to true retention by taking into account weight yield factors [23], which provided a more accurate means of comparing nutrient loss or gain.

## Conclusions

In general, nutrient true retentions for the five selected spices were explored using seven different cooking procedures. Different true retentions are probably due to food types, the cooking procedures, and interactions between the two. The major observation regarding nutrient true retention was that deep-frying and stir-frying better retained nutrients and steaming showed lower true retention values for selected spices (i.e., crown daisy, unripe hot pepper, onion, garlic, and red pepper). As different culinary treatments have different effects on particular vegetable, the use of suitable cooking methods could enhance the nutrition values of cooked foods and improve overall acceptability. More investigations on the spices from different locations and harvested at different times are necessary. Also, further studies will be undertaken to comprehensively elaborate the true retention of different nutrients of spices with other ingredients.

## Supporting information

S1 Data Supporting information. (XLSX)
